# Development and establishment of a patient advisory board for forensic psychiatric patients - Insights and experiences from the PART project

**DOI:** 10.1192/j.eurpsy.2024.1206

**Published:** 2024-08-27

**Authors:** E. Drewelow, M. Daum, F. Ferra, K. Gerullis, I. Kilimann, O. Klein, P. Walde, S. Teipel, B. Völlm

**Affiliations:** ^1^Clinic for Forensic Psychiatry; ^2^Clinic for Psychosomatic Medicine and Psychotherapy, Rostock University Medical Center; ^3^(DZNE), Deutsches Zentrum für Neurodegenerative Erkrankungen, Rostock, Germany

## Abstract

**Introduction:**

Participatory research (PF) actively involves people with lived experience (pwle), e.g. for a disease, in research. This improves the relevance, quality and impact of research and can help to raise third-party funds, increase recruitment numbers, select research methods. Pwle can support all stages of the research process, including dissemination. While PF is already standard in other countries, Germany is still lagging behind. Our participatory advisory board aims to create a sustainable structure to involve underrepresented patients.

**Objectives:**

In the PART advisory board, pwle and researchers should actively cooperate in projects in the field of forensic psychiatry. In preparation to establish the advisory board procedures, key documents and training material were developed. In addition experiences, opinions, ideas and concerns of stakeholders and pwle in relation to PF were collected.

**Methods:**

Guided interviews were conducted with stakeholders (clinical, research) and focus groups with in-patient pwle from forensic psychiatry. They were asked how they imagine the structure, tasks and goals of a participatory advisory board, what opportunities and obstacles they see. Anticipated framework conditions and support needs for the successful implementation were also asked. The interviews and focus groups were audio-recorded and transcribed. Data was analysed with MAXQDA using thematic analysis.

**Results:**

In total, 8 expert interviews and 2 focus groups with 15 pwle were conducted in the first half of the year 2023. The analysis so far shows great interest in PF, although the term is mostly unknown and experience seems to be limited. The respondents identified opportunities for participatory research, but also challenges that need to be overcome in terms of its implementation. Both groups emphasise the importance of PF, especially in the field of mental illness, and express ideas for its implementation.

**Conclusions:**

The results will be incorporated into the structure of the advisory board, so that PF in the field of forensic psychiatry will be more successful and the exchange between researchers and pwle will be facilitated. Detailed results as well as impressions from the first meeting(s) of the advisory board will be presented at the EPA conference.

**Disclosure of Interest:**

None Declared

